# Resistance to thyroid hormone β in autoimmune thyroid disease: a case report and review of literature

**DOI:** 10.1186/s12884-018-2110-9

**Published:** 2018-12-03

**Authors:** Di Wu, Rui Guo, Huiling Guo, Yushu Li, Haixia Guan, Zhongyan Shan

**Affiliations:** Department of Endocrinology and Metabolism, The Endocrine Institute and The Liaoning Provincial Key Laboratory of Endocrine Diseases, The First Hospital of China Medical University, 155 Nanjing Bei Street, Shenyang, Liaoning 110001 People’s Republic of China

**Keywords:** Resistance to thyroid hormone, Autoimmune thyroid disease, Gestational monitoring

## Abstract

**Background:**

Resistance to thyroid hormone beta (RTHβ) results in symptoms of both increased and decreased thyroid hormone action. The effect of thyroid hormone changes in different types of autoimmune thyroid disease (AITD) in RTHβ is dynamic.

**Case presentation:**

A 25-year-old Asian female had a RTHβ Y321C mutation with Hashimoto’s thyroiditis and type 2 diabetes mellitus. She was followed-up through gestation and two years postpartum, revealing development of postpartum thyroiditis (PPT) with characteristic wide fluctuations in serum thyrotropin levels, and of spontaneous recovery from an episode of transient hypothyroidism. The presence of RTHβ did not prolong thyroiditis duration nor progressed toward permanent hypothyroidism. Prenatal genetic analysis was not performed on the unaffected fetus, and did not result in congenital hypothyroidism, possibly because maternal free thyroxine (FT4) levels were mildly elevated at less than 50% above the reference range in early gestation and gradually decreased to less than 20% after the 28th gestational week.

**Conclusion:**

In RTHβ patients with autoimmune thyroid disease, episodes of thyroid dysfunction can significantly alter thyrotropin levels. During pregnancy, mildly elevated maternal free thyroxine levels less than 20% above the upper limit may not be harmful to unaffected fetuses. Unnecessary thyroid hormone control and fetal genetic testing was avoided during the gestational period with monthly follow-up.

## Background

Resistance to thyroid hormone (RTH) is a rare autosomal dominant condition of elevated free thyroxine (FT4) and free triiodothyronine (FT3) with non-suppressed thyrotropin (TSH) levels due to reduced sensitivity to thyroid hormone. Mutations are mostly heterogeneous in the thyroid hormone receptor α or β gene causing symptoms of both increased and decreased thyroid hormone action, depending on the tissues’ predominant receptor isoform expression, the magnitude of hormonal resistance, and the effectiveness of compensatory mechanisms. RTHβ accounts for approximately 90% of RTH in which patients manifest goiter and tachycardia [1].

Autoimmune thyroid disease (AITD) is defined by the presence of thyroid autoantibodies, which is common in the general population. Graves’ disease and Hashimoto’s thyroiditis (HT) are forms of AITD that can cause hyperthyroidism and hypothyroidism. Physiological and immunologic changes during pregnancy and postpartum period can ameliorate and aggravate thyroid immunity, respectively, leading to postpartum thyroiditis (PPT). Transient thyroid dysfunction from AITD feedback to the impaired thyroid hormone receptors in RTHβ [2].

We present a case of RTHβ with concomittent AITD in whom the natural thyroid hormone profile was followed for 4 years, including through pregnancy and during the postpartum period. We also review the current literature on the management and outcomes of cases of RTHβ and AITD in pregnancy.

## Case presentation

A 25-year-old female presented with heat intolerance, palpitations, weight gain, and goiter. Thyroid function tests showed FT4 at 24.46 pmol/L (normal range, 9.01–19.05), FT3 at 7.31 pmol/L (normal range, 2.63–5.70), and TSH at 8.63 mIU/L (normal range, 0.35–4.94). Radioiodine uptake was 21.95% at 3 h (normal range, 10–30%) and 41.5% at 24 h (normal range, 25–60%). TSH was stimulated from 4.50 to 34.40 mIU/L 15 min after intravenous bolus of thyrotropin-releasing hormone (TRH); liothyronine (L-T3) suppressed TSH from 4.61 mIU/L to 0.21 mIU/L [3]. Pituitary magnetic resonance imaging (MRI) revealed no abnormality. Gene sequencing identified a heterozygous Y321C substitution mutation in exon 9 of the *THRB* gene [4], thereby confirming the diagnosis of RTHβ. Dual-energy X-ray absorptiometry (DEXA) scan revealed decreased bone mass. The patient was considered as susceptible to Hashimoto’s thyroiditis based on positive thyroid peroxidase antibodies (TPOAb), positive thyroglobulin antibodies (TgAb), and negative thyrotropin receptor antibodies (TRAb). She was given L-T3 to suppress TSH, and a β-blocker to manage tachycardia.

In addition, she had a history of diabetes mellitus without glucose management that resulted in hemoglobin A1c (HbA1c) at 7.1%; oral glucose tolerance test (OGTT) showed basal glucose 7.8 mmol/L (15.2 mmol/L at 120′) and basal insulin 19.47 mIU/L (80.87 mIU/L at 120′), and homeostasis model assessment-estimated insulin resistance (HOMA-IR) was 6.75. Her body mass index (BMI) was 28.3 kg/m^2^ at 155 cm height. She had liver steatosis and serum triglyceride level was 3.05 mmol/L.

**Fig. 1:**
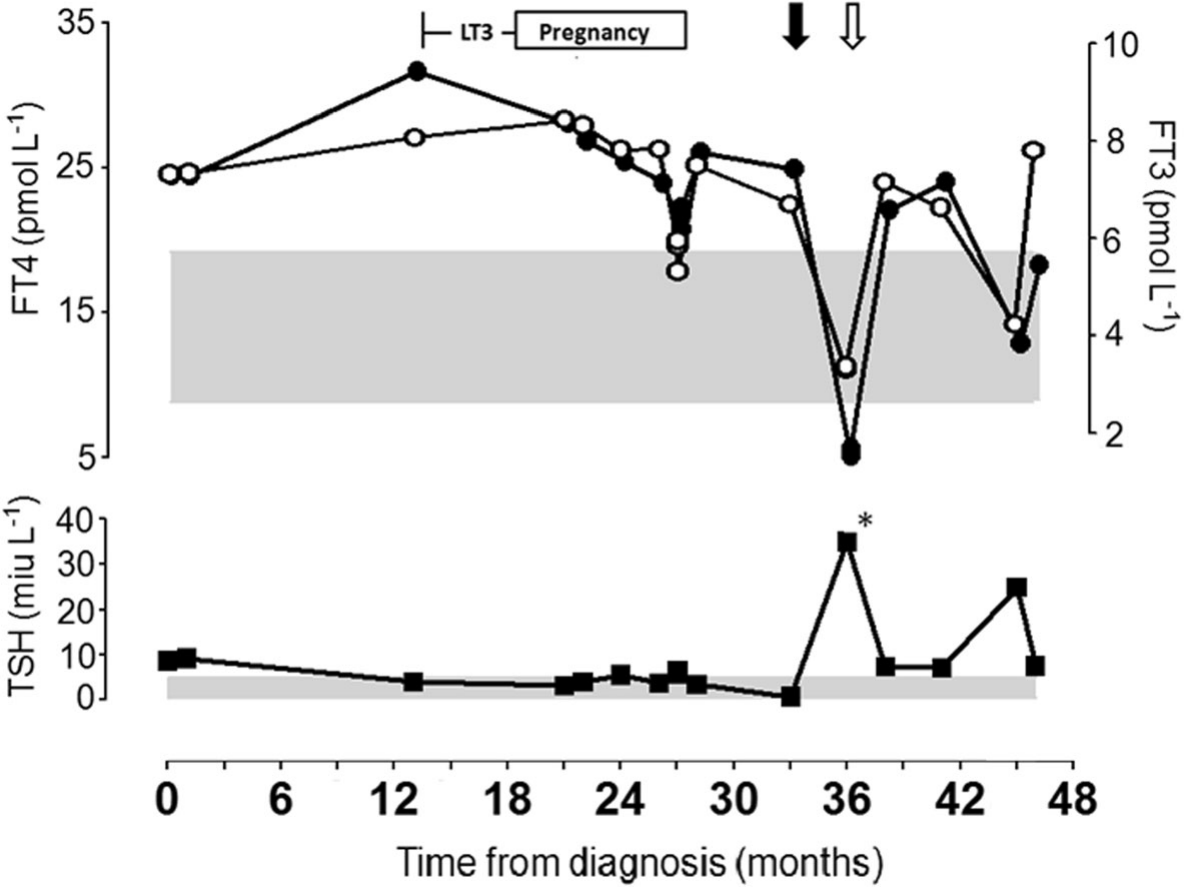
Changes in thyroid function. FT4 (black circle), FT3 (white circle), and TSH (black square) were monitored in the patient diagnosed with RTHβ and autoimmune thyroiditis. The thyrotoxic phase of postpartum thyroiditis occurred 6 months after delivery (solid black arrow), followed by a hypothyroid phase at 9 months (white arrow), and the return to *status quo ante* at 11 months postpartum. Hypofunctioning thyroid from Hashimoto’s thyroiditis occurred at 18 months postpartum, followed by spontaneous recovery after 1 month. * Indicates TSH values exceeding the instrument’s upper limit: TSH > 100 mIU/L. Abbreviations: LT3, levotriiodothyronine therapy; FT4, free thyroxine; FT3, free triiodothyronine; TSH, thyrotropin

The patient requested ovulation induction after 2 years of irregular menstruation and infertility. The singleton pregnancy was confirmed at 13 weeks gestation, then both LT3 and β-blocker were discontinued. Her thyroid functions and fetal ultrasound morphology were monitored every 1–4 weeks (more frequently during the initial and last month), and revealed no complications. Based on the mild elevation of maternal thyroid hormones and patient preference, fetal *THRB* gene testing was not performed. The patient also did not require propranolol or propylthiouracil during pregnancy for RTH because she was asymptomatic and had mildly elevated and stable thyroid functions (Fig. 1). Since FT4 crosses the placenta, propylthiouracil is recommended by the Endocrine Society to decrease FT4 levels if it is too high. L-T3 was not considered since it does not cross the placenta. However, she did require strict glucose control for previously diagnosed diabetes mellitus with insulin titrated to 52 units daily towards the end of gestation (HbA1c 5.20–5.50%). She was instructed to closely monitor her glucose levels, and she did not experience any hyper- or hypoglycemic symptoms or emergencies during pregnancy. The pregnancy was uneventful until premature rupture of membranes at 37 weeks, which developed to placental abruption during observation, and a healthy neonate of 3210 g was delivered by caesarean. The newborn did not have a *THRB* mutation. Suppressed TSH and low birth weight commonly seen in unaffected infants born to RTH mothers was not observed. The newborn did not show signs of thyroid dysfunction in the follow-up: FT4 was 14.47 pmol/L with TSH 5.05 mIU/L at one-month-old, and FT4 12.28 pmol/L with TSH 1.20 mIU/L at one-year-old. After delivery, the patient was restarted on L-T3 (low starting dose for titration), and insulin (24–26 units daily) doses were readjusted, but all medications were discontinued 1 month after delivery due to poor compliance.

At 6 months postpartum, the patient showed marked suppression of TSH compared with baseline (0.59 mIU/L vs. 8.63 mIU/L) with elevated FT3 (6.70 pmol/L) and FT4 (24.94 pmol/L), but remained clinically euthyroid. HOMA-IR was 7.97 with fasting glucose at 10.12 mmol/L. Suppressed TSH in RTH followed by exceedingly elevated TSH (> 100 mU/L) at 9 months postpartum indicated the occurrence of PPT, which recovered at 11 months postpartum (Fig. 1). Fasting glucose and insulin varied between 8 and 12 mmol/L and 17.5–21.8 mIU/L, respectively. HOMA-IR during the hypothyroid phase could not be calculated from medical charts. Glycemia was managed with diet and exercise, though controlled less ideally compared to prepregnancy.

At 18 months postpartum, the patient was asymptomatic apart from discomfort due to a III degree goiter. TSH were again elevated, but recovered spontaneously after 1 month suggesting an episode of Hashimoto’s thyroiditis. No medication was prescribed for this period.

The index member’s biological mother had a history of poorly-controlled Graves’ disease, and diabetes mellitus. She was later found to carry the same *THRB* mutation. *THRB* gene sequencing of the index case’s spouse and father were negative for mutation. Second-degree relatives reported no symptoms related to thyroid dysfunction, and thyroid function screening was normal without inappropriate TSH secretion, however, they declined gene sequencing.

## Discussion and conclusions

We observed the natural fluctuations in thyroid hormone levels of RTHβ under the influence of gestational physiology and autoimmune thyroiditis. Table 1 reviews the literature of the management and outcomes of RTHβ pregnancies with and without AITD.

**Table 1:** Literature review of RTHβ and AITD in pregnancy

Author	RTHβ THRB mutation	AITD Thyroid antibodies	Gestational management for RTHβ	Maternal outcome	Foetal outcome
Jonas et al. 2014 [5]	R320C (c1243C > T)	Hashimoto’s thyroiditis: TPOAb and TgAb positive, TRAb negative.	3 pregnancies. Levothyroxine (LT4) to maintain an fT4 range within 20% of the upper limit of normal. No chorionic villous sampling.	Uneventful pregnancies. Developed postpartum thyroiditis (PPT).	Full-term neonates had normal birth weights and body lengths. The first child harboured the THRB mutation.
Paragliola et al. 2013 [10]	V283A (g.361470 T > C) on exon 8	TPOAb and TgAb positive, TRAb negative.	2 pregnancies. Monthly monitoring of her thyroid function. Did not require any treatment	Uneventful pregnancies. Developed PPT after pregnancies.	Full-term male neonates with normal birth weights and normal thyroid function.
Boix et al. 2007 [11]	M310 V on exon 9	Iatrogenic hypothyroidism after radioiodine treatment for autoimmune hyperthyroidism	Triiodothyronine (LT3) to maintain normal TSH. Unsuccessful amniocentesis for RTH testing.	Uneventful pregnancy	36 week unaffected male neonate with normal birth weight.
Furlanetto et al. 2000 [12]	M310 L (928A > T) on exon 9	TPOAb negative.	–	Uneventful pregnancy.	Full-term affected female neonate with length 46 cm (5–10th percentile), and weight 2250 g (<5th percentile). Elevated thyroid function.
Sarkissian et al. 1999 [13]	T329 N on exon 9	Antithyroid, anti-T4 and anti-T3 antibodies were negative.	Dextrothyroxine (DT4) therapy was discontinued upon pregnancy.	Five spontaneous abortions.	35 week unaffected male neonate with normal weight and normal thyroid function. 38 week affected female neonate weight 2850 g and length 47 cm (height/Age at -2SD compared to -1SD in both parents, and body mass index of 12.9 at the fifth percentile for age).

Abbreviations: *TPOAb* peroxidase antibodies, *TgAb* thyroglobulin antibodies, *TRAb* TSH receptor antibodies, *fT4* free thyroxine

Unnecessary thyroid medication was avoided during the gestational period with frequent 1–4 week follow-ups in our patient. However, no recommendations has been made to suggest even more frequent follow-up intervals are needed for RTH mothers to unaffected fetuses [3]. On the other hand, the Endocrine Society recommends that FT4 levels should be maintained at less than 20% above the reference range in RTH mothers carrying unaffected fetus [3]. Although the FT4 levels in our patient reached the recommended levels only after the 28th gestational week, this did not result in congenital hypothyroidism in the unaffected fetus, possibly because maternal FT4 levels were mildly elevated at less than 50% above the reference range in early gestation and gradually decreased throughout pregnancy. In addition, the value of fetal gene analysis was small as maternal thyroid hormone levels gradually decreases in pregnancy, thus making the fetal growth environment reasonably less toxic. This decline in thyroid hormone levels during pregnancy is common, and should be taken into account for affected mothers to avoid overtreatment. Lack of prenatal genetic analysis on the unaffected fetus did not result in congenital hypothyroidism in our patient and other reported cases [5]. RTHβ did not prolong PPT or transient thyroiditis disease duration or progression toward permanent hypothyroidism.

Barkoff et al. [6] determined an increased prevalence of AITD among RTH-β patients, and postulated that susceptibility to AITD may be due to an underlying genetic disposition. Our subjects have shown that in first-degree relatives the variation in type of AITD phenotype under RTH-β is present; RTH-β with HT, and RTH-β with GD. Moreover, the natural progression of AITD is independent of inherent RTH, the development of PPT and spontaneous recovery from follicular damage in HT, the presence of RTH-β did not increase disease duration or progression toward permanent hypothyroidism.

Another observation is that glucose metabolism is independent of TFT results, in this case family, DM was diagnosed in the two members with genetically-confirmed RTH-β. Currently, the prevalence of DM in RTH patients is unknown. Apart from significantly higher insulin resistance and lower insulin sensitivity in RTH-β patients than control subjects as observed by Mitchell et al. [7], the relationship between TH and blood glucose in RTH-β remains undetermined. In our patient, the most dramatic change in TH occurred during the hypothyroid phase of PPT (Fig. 1), at which basal glucose and insulin was still comparable to baseline levels, indicating that RTH-β may also blunt the effect of significant TH fluctuation on insulin resistance. Furthermore, the management of one condition might be able to alleviate another. Stagi et al. [8] proposed that TRIAC therapy might alleviate hyperinsulinism and improve insulin resistance after one year. Although it is unclear whether these drug responses are clinically significant in the majority of RTH-β patients [9], it is important to note that the extent of dominant-negative effect in mutant TH receptors and the degree of islet cell dysfunction in diabetes disease course for each individual is different.

This case report highlights an important issue of whether initiating pharmacologic treatment in RTHβ condition is always needed during pregnancy. In cases where FT4 levels are mildly elevated and decreasing throughout pregnancy and where prenatal genetic testing is not performed, it is advisable to adopt an attentive and cautious monitoring and care. This case report supports that conservative approach can have a positive outcome without harming the RTHβ mother and the unborn unaffected fetus.

## Abbreviations

AITD: Autoimmune thyroid disease
DEXA: Dual-energy X-ray absorptiometry
FT3: Free triiodothyronine
FT4: Free thyroxine
HT: Hashimoto’s thyroiditis
L-T3: Liothyronine or 3,5,3’-Levo-triiodothyronine
MRI: Magnetic resonance imaging
PPT: Postpartum thyroiditis
RTH: Resistance to thyroid hormone
TgAb: Thyroglobulin antibodies
TPOAb: Thyroid peroxidase antibodies
TRH: Thyrotropin-releasing hormone
TSH: Thyrotropin, or thyroid stimulating hormone
